# Validation and Administration of the Spanish Questionnaire ‘Humanisation of Pediatric Care in Pain Management with a Non-Pharmacological Approach (HUPEDCARE-Q)’

**DOI:** 10.3390/children12081036

**Published:** 2025-08-07

**Authors:** Inmaculada García-Valdivieso, Jorge Sánchez-Infante, Miriam Hermida-Mota, Sonsoles Hernández-Iglesias, Pablo Pando Cerra, Sagrario Gómez-Cantarino

**Affiliations:** 1 Faculty of Physiotherapy and Nursing, University of Castilla-La Mancha, 45071 Toledo, Spain; inmaculada.garciavaldivieso@alu.uclm.es (I.G.-V.); miriam.hermida@alu.uclm.es (M.H.-M.); sagrario.gomez@uclm.es (S.G.-C.); 2 Faculty of Health Sciences, Francisco de Vitoria University, 28223 Madrid, Spain; jorge.sanchezinfante@ufv.es; 3 Physiotherapy Research Group of Toledo (GIFTO), Faculty of Physiotherapy and Nursing, University of Castilla-La Mancha, 45071 Toledo, Spain; 4 Department of Construction and Manufacturing Engineering, University of Oviedo, 33203 Asturias, Spain; pandopablo@uniovi.es; 5 Health Science Research Unit, Nursing (UICISA: E), Coimbra Nursing School (ESEnfC), 3004-011 Coimbra, Portugal

**Keywords:** nursing education, pain management, validity, reliability, nursing research, pediatric pain, nursing care, non-pharmacological interventions

## Abstract

**Background/Objectives**: The pain associated with invasive procedures is one of the most common experiences in the pediatric population. Its management remains insufficient due to gaps in healthcare training and knowledge. The aim of this study was to analyze the attitudes, beliefs, care practices, and training of healthcare professionals in relation to pediatric pain, through the development and application of the questionnaire ‘Humanisation of Pediatric Care in Pain Management with a Non-Pharmacological Approach (HUPEDCARE-Q)’. **Methods**: A cross-sectional, observational, and descriptive study with a quantitative approach was conducted to validate a questionnaire. The process was carried out in three phases: (1) design and initial development of the instrument; (2) evaluation of content validity through expert judgment, using the Content Validity Coefficient (CVC); (3) administration of the questionnaire to a large sample of healthcare professionals to assess its internal consistency and psychometric structure. **Results**: The evaluation involved five experts, and the items were assessed using the Content Validity Coefficient (CVC), with the overall CVC of the questionnaire exceeding 0.80. The average item scores given by the experts ranged from 0.88 to 0.95, indicating a high level of agreement in their evaluations. The results showed statistically significant positive correlations among most items (*p* < 0.001), indicating adequate internal consistency. **Conclusions**: The content validation and pilot study confirmed the theoretical relevance and appropriateness of the HUPEDCARE-Q questionnaire items in the Spanish context. The results support its usefulness as a valid and reliable tool to identify attitudes, beliefs, knowledge, and training needs in the humanized management of pediatric pain.

## 1. Introduction

The role of nursing in pediatric units has evolved over time, occupying a privileged position that allows it to be at the forefront of care, serving as a driving force for innovation and improvement in healthcare [1,2]. Nursing care in these units involves combining aspects derived from new technologies with the human dimension, due to the direct and continuous contact established with patients and their families [3,4].

In the early stages of life, children have difficulty expressing the pain they experience, making it essential for healthcare professionals to recognize the signs in order to relieve this discomfort in the most appropriate way [5,6,7]. Humanizing pediatric care involves viewing the child as a biopsychosocial being, avoiding an approach that focuses solely on pathological and procedural aspects [8]. Pain resulting from invasive procedures is one of the most common types among the pediatric population [9]. Among the most frequent interventions are heel pricks for screening endocrine/metabolic diseases, peripheral venous catheterization, blood draws, and vaccine administration [10,11].

Several studies show a lack of knowledge among healthcare professionals regarding pain, its assessment, and treatment [12,13,14]. Health sciences students also lack the necessary knowledge and skills to perform a comprehensive clinical assessment and manage pain appropriately [15]. Globally, the development of interdisciplinary pain education during the pre-professional stage of university training is being promoted [16]. The aim is to improve students’ understanding of pain mechanisms and related biopsychosocial concepts, as well as to foster better collaboration and communication among the different professions involved in pediatric care [17,18]. For this reason, it is necessary to use new methods to measure healthcare professionals’ knowledge about pediatric pain, it is prevention, and proper management, in order to promote ongoing education and provide more sensitive, tailored care for pediatric patients [19,20].

There are several validated assessment instruments, such as the Pediatric Nurses Knowledge and Attitudes Survey Regarding Pain (PNKAS). This questionnaire evaluates nurses’ knowledge and attitudes regarding pediatric pain management [21]. It has been used in various studies to identify areas in need of improvement in healthcare staff training. The survey includes specific items on pain in newborns, children, and adolescents, as well as modified dosing related to pharmacological aspects [22,23,24]. On the other hand, the Nurses Knowledge and Attitudes Survey Regarding Pain (KASRP) examines different aspects related to pain assessment, therapeutic approaches (both pharmacological and non-pharmacological), possible adverse effects of medications, and interdisciplinary collaboration in the comprehensive care of patients experiencing pain [25,26]. Therefore, this study aimed to develop and validate an instrument designed to assess the attitudes, beliefs, care practices, and training of current and future healthcare professionals. The purpose of this tool is to identify potential knowledge gaps among care staff regarding the identification of pediatric pain, as well as the alternative treatments available for its prevention and management.

Although there are validated instruments to assess pediatric pain, it is believed that a tool completed based on caregivers’ perceptions regarding the needs and care strategies for children, shaped by their academic training, practical experience, and educational resources, may enhance the humanization of pediatric pain management [27]. In this way, the questionnaire becomes a useful assessment tool to measure knowledge, attitudes, and beliefs applicable to healthcare. Likewise, an instrument completed by healthcare professionals is considered reliable, as they work in specific care settings and have a greater influence on the child’s development [28].

The development and validation of instruments is essential for organizing, planning, and evaluating healthcare behavior in response to pediatric pain and the interventions for its prevention. Therefore, it is crucial to pay close attention to the process of developing and validating an instrument. Questionnaires become key tools for data collection and are especially effective in supporting professionals in their caregiving roles [29].

## 2. Materials and Methods

This is a cross-sectional, observational, methodological, and descriptive study with a quantitative approach, which was carried out in three phases: (1) development of the first version of the “Humanisation of Pediatric Care in Pain Management with a Non-Pharmacological Approach (HUPEDCARE-Q) Questionnaire”; (2) content validation by a panel of experts; and (3) administration of the revised questionnaire to healthcare professionals in a pilot study with the target audience. The study was approved by the Social Research Ethics Committee of the University of Castilla-La Mancha (UCLM), Spain, under registration number CEIS-2025-91937.

### 2.1. Participants

Seven health professionals, including specialists in maternal and child health, were invited to participate in the study as judges during the content validation phase. However, only five returned the requested responses. One of the judges is a pediatrician who has been working in Specialized Care for 12 years. The second is a physiotherapist with 15 years of experience in the field of pediatrics. The third judge is a professional who has completed a specialization in pediatric nursing and has worked in Primary Healthcare for 11 years. The fourth judge is a nurse manager in the maternal and child health area of Specialized Care, with 25 years of experience. The fifth judge is a university professor specializing in women’s and children’s health, with 19 years of experience in projects related to the study area.

The target population of the pilot study consisted of practicing healthcare professionals and health sciences students over the age of 18. Participants included those enrolled in university programs at public or private institutions (such as Nursing, Medicine, Physiotherapy, or related degrees), as well as active professionals working in Primary Care, Specialized Care, or private pediatric healthcare centers. The selection of this population was based on their current or future involvement in the care of pediatric patients and, consequently, their potential influence of pediatric pain management. Their inclusion was essential for assessing the level of knowledge, beliefs, attitudes, and practices related to pediatric pain from a multidisciplinary and humanized perspective.

### 2.2. Procedures, Instruments, and Data Collection

The questionnaire for evaluating pediatric care in pain management using a non-pharmacological approach (HUPEDCARE-Q) was developed to assess the practices and actions of healthcare professionals regarding pediatric pain. The questionnaire was structured into four sections, with the first dedicated to collecting sociodemographic data. The second thematic block aimed to explore attitudes and beliefs about pediatric pain. This section was developed based on a review of scientific articles that examined the comparison of different perspectives and beliefs among healthcare professionals regarding the management of children’s pain [30,31,32,33].

The third thematic block assessed knowledge and healthcare training related to pediatric pain, based on a literature review and focused on understanding pediatric pain, educational levels, and teaching methods within the Health Sciences [31,34]. The final section of the questionnaire consisted of 10 items on best practices in pediatric pain management, presented as statements or denials regarding cognitive, behavioral, and emotional aspects in response to pediatric pain as perceived by the child [35,36,37].

All items were developed with the consideration that healthcare professionals should be able to assess their own knowledge on an individual level regarding specific actions performed in clinical practice, thereby enhancing their understanding of pediatric pain. The second thematic block included 11 questions, and the third block included 7, with responses presented on a Likert scale ranging from 0 (strongly disagree) to 5 (strongly agree). The total score from this questionnaire yields a result that is interpreted as the healthcare professional’s performance in managing pediatric pain. The fourth thematic block consisted of 10 questions, which were structured so that a NO response equaled 0, and a YES response equaled 1. A score with five or more YES responses suggests a higher level of preparation, responsiveness, and sensitivity in managing pediatric pain. This implies a more appropriate and patient-centered care approach by healthcare professionals for this specific population.

The final score was obtained by summing the numbers corresponding to each response option selected by each participant for each item. In other words, each response option was assigned five scores, with the minimum total score ranging from 0 to 28 and the maximum reaching 100. A higher score indicates better performance in the assessment and management of pediatric pain, as well as a greater integration of practical training and the use of appropriate tools. In this pilot study, a score below 50 was considered to reflect a negative impact, indicating a greater lack of attitudes, beliefs, knowledge, training, and best practices regarding pediatric pain management.

In relation to the Attitudes and Beliefs block, the need to individualize analgesic treatment according to the patient’s response is also analyzed, as well as the convenience of the combined use of pharmacological and non-pharmacological methods. The biopsychosocial dimension of infant pain is also considered, considering biological, psychological, and social factors in its perception and expression. The efficacy of non-pharmacological interventions such as breastfeeding, non-nutritive sucking, use of sucrose, among others, is highlighted, especially in cases of mild to moderate pain. Other important aspects include parental presence during painful procedures, the use of placebos and pain tolerance as indicators of pain authenticity, and the possible risk of opioid addiction in pediatric pain.

The section Knowledge and Training in pediatric pain is designed to assess participants theoretical and practical training in relation to the identification and management of pediatric pain. Various key aspects are analyzed to evaluate not only the level of existing knowledge but also the effective application of tools and protocols in the clinical context. The overall objective of this section is to comprehensively assess the professionals’ level of training in the field of pediatric pain, identifying strengths and potential shortcomings that can be addressed through more specific and up-to-date training programs.

The dimension Best Practices: Pediatric Pain Management addresses the perceived adequacy of training received by healthcare professionals in identifying, evaluating, and managing acute pediatric pain effectively. The ultimate goal is to determine whether clinical practice reflects a humanized, child-centered approach that prevents both immediate distress and long-term psychological or developmental sequelae.

### 2.3. Content Validation with Experts in the Field

For the content validation process, it is recommended that the questionnaire be evaluated by experts in the theoretical and practical knowledge area of the instrument, with a minimum of 3 to 5 judges [38]. In this case, an electronic invitation was sent to 7 healthcare professionals in the pediatric field to participate in the validation process. Along with the invitation, an evaluation form, a brief theoretical reference on the topic, and an explanation of the evaluation process were included. Five of the professionals responded and submitted their evaluations within the stipulated timeframe, thus meeting the minimum criteria established for the panel of judges [38].

Each expert evaluated the items in each block of the questionnaire (2nd, 3rd, and 4th blocks) according to three criteria: Clarity of language, Pertinence, and Relevance of the questions. They assigned a value from 1 to 4 based on their understanding of the content expressed in each item, where 1 = Very little; 2 = Little; 3 = Much; 4 = Very much.

The following guiding questions were used to assess clarity: “Is the language of each item sufficiently clear and understandable for the study participants? Will the participants understand this item without ambiguity?”

For pertinence, the question was: “Do you consider the items presented to be pertinent for evaluating the specific aspect intended to be measured in the survey?”

Finally, for relevance, experts were asked: “Are the proposed items relevant to the context and target population?”

And the end of the evaluation, experts were able to provide comments, opinions, suggestions, and proposed adaptations to any element of the instrument. They could also suggest the addition of a new item/question or the elimination of an existing one.

### 2.4. Questionnaire Administration to Healthcare Professionals

This phase of the research involved a pilot study to apply the proposed instrument to the target audience (students and healthcare professionals in the field of pediatrics). Participants received a link to complete the questionnaire online (HUPEDCARE-Q). To respond to the survey, participants needed an electronic device with internet access. The first part of the questionnaire included an Informed Consent form, outlining the protection of personal data and the assurance of digital rights. It also provided information about the research and emphasized the voluntary and anonymous nature of participation. Additionally, participants were informed that they could withdraw from the study at any time if they wished.

The target audience was invited to participate in the research through social media, official organizations, professional associations, the project’s own website, participating universities, and various groups. Additionally, participants were encouraged to contact the researchers if they had any questions while completing the questionnaire. The collected data were stored in an electronic spreadsheet and kept confidential, in accordance with ethical principles for research involving human subjects, and were used solely for the development and publication of this article. Participants were informed that the data would be used until the publication date or for a maximum of five years. This phase was carried out between October and December 2024.

### 2.5. Statistical Analysis

Prior to administering the questionnaire, its content validity was assessed using the Content Validity Coefficient (CVC) to determine whether the questions aligned with their intended objectives, based on the analysis and agreement of a panel of five expert judges in pediatric pain, physiotherapy, and research methodology. Each item was evaluated in terms of its clarity, pertinence, and relevance to the corresponding dimension.

To quantify the level of agreement among the evaluators, the CVC values proposed by Hernández-Nieto (2002) were calculated, including the individual CVC (CVC_i_), the CVC by dimension (CVC_d_), and the total CVC of the instrument (CVC_t_) [39]. CVC values ≥ 0.80 were considered acceptable. The estimation error for each item (Pei) was also calculated in order to refine the instrument prior to its psychometric analysis.

Data analysis was performed using IBM SPSS Statistics software (version 24). The internal consistency of each questionnaire dimension was assessed using Cronbach’s alpha coefficient, with values ≥ 0.70 considered acceptable [40]. To explore the internal structure of the instrument, an exploratory factor analysis (EFA) was conducted using the principal component extraction method and Varimax rotation [41].

The suitability of the data for EFA was verified using the Kaiser–Meyer–Olkin (KMO) index and Bartlett’s test of sphericity [42]. Factor loadings greater than 0.40 were considered adequate. Descriptive measures (mean and standard deviation) were calculated for each dimension, and correlations between dimensions were analyzed using Pearson’s correlation coefficient. The level of statistical significance was set at *p* < 0.05.

## 3. Results

### 3.1. Content Validity Analysis

The content validity analysis of the items included the calculation of the CVC and its corrected version (CVCpei), based on the evaluation of five experts. Each expert assessed the clarity, relevance, and appropriateness of the items in relation to the instrument’s two dimensions. The results obtained for each item, as shown in Table 1, are as follows:

‐ CVC by item: All items showed a CVC greater than 0.80, indicating that they are highly representative of the dimensions they assess.
‐ Corrected CVC (CVCpei): After adjusting for the judges’ error (Pei), the corrected CVC values for all items remained above 0.80, confirming the content validity of the items.
‐ Total CVC (CVCt): The total CVC for the instrument, obtained as the average of the individual values, also exceeded the 0.80 threshold, indicating that the instrument as a whole has adequate validity.

The average scores given by the experts per item ranged from 0.88 to 0.95, indicating a high level of agreement in the evaluations. Additionally, the corrected CVCpei values were consistent, reinforcing the validity and stability of the evaluated items. Therefore, the results of the content validity analysis confirm that the items of the instrument are appropriate and relevant to the dimensions they are intended to measure, with a high degree of agreement among the evaluators (Table 1 and Table 2).

### 3.2. Internal Consistency

The internal reliability, measured using Cronbach’s alpha coefficient, was adequate in both dimensions. For the final part of the questionnaire, Cronbach’s alpha coefficient was adequate for the dimension consisting of 10 dichotomous YES/NO questions (Table 3).

### 3.3. Exploratory Factor Analysis

The sampling adequacy analysis yielded a KMO index of 0.90, indicating good suitability for EFA. Bartlett’s test of sphericity was significant (χ^2^ (153) = 6650.48, *p* < 0.001), confirming the appropriateness of the data. The factor analysis extracted two factors with eigenvalues greater than 1, which explained 57.3% of the total variance (Table 4).

Additionally, a Pearson correlation analysis was conducted between the 18 items of the instrument to explore the relationship between the measured variables. The results showed statistically significant positive correlations between most of the items (*p* < 0.001), indicating adequate internal consistency. In particular, moderate to high correlations were observed between items 13 and 14 (r = 0.808), as well as between items 13 and 17 (r = 0.600), and items 14 and 16 (r = 0.594), suggesting a strong conceptual association between these items. In contrast, the lowest correlations, though still significant, were found between certain items such as P3 and P5 (r = 0.114), indicating a weaker relationship between these aspects of the evaluated construct. Overall, these findings support the convergent validity of the instrument.

For the second part of the questionnaire, the sampling adequacy analysis yielded a KMO index of 0.70, indicating good suitability for EFA. Bartlett’s test of sphericity was significant (χ^2^ (153) = 2073.74, *p* < 0.001), confirming the appropriateness of the data. The factor analysis extracted one factor with eigenvalues greater than 1, which explained 63.55% of the total variance (Table 5).

The sample consisted of 1.120 healthcare professionals (83.50% women, 16.50% men; 78% were between 20 and 30 years of age). The questionnaire items showed mean scores ranging from 1.56 to 4.17, with standard deviations between 1.19 and 1.64 on the Likert scale. No significant outliers or skewness were observed in the distributions. Professionals demonstrated moderate to low preparedness in pediatric pain management, with high emotional sensitivity and a positive willingness to improve. Responses were consistent, indicating good comprehension of the instrument. (Table 6 and Table 7).

## 4. Discussion

This study aimed to develop and validate the content of a questionnaire designed to assess the attitudes, beliefs, training, level of knowledge, and best practices of future and current health professionals. It also addressed the management of pediatric pain through non-pharmacological treatments, within the framework of the HUPEDCARE-Q project. The instrument was designed to collect information based on experiences, practices, and perceptions, with the goal of identifying strengths and gaps in infant pain care, especially in healthcare settings.

The content validation process of the HUPEDCARE-Q demonstrated a solid theoretical foundation, and its items proved to be relevant and appropriate for the Spanish healthcare context. The language used in this tool is accessible to professionals from various fields within the health and social sectors, trained in the comprehensive approach to pediatric pain, including its care, assessment, and management. Therefore, the instrument is considered to have content validity and is suitable for use in evaluating attitudes, beliefs, training, knowledge, and best practices in pediatric pain management.

It is important to highlight that this questionnaire is not intended to provide clinical diagnoses; rather, it should be used as a tool for collecting information, which can then be analyzed and interpreted by specialists in the field of pediatric pain. The sample consisted of 1.120 healthcare professionals, of whom 83.50% were women and 16.50% men. Among the respondents who participated in the validation of the HUPEDCARE-Q on pediatric pain and its non-pharmacological management, 78% were between 20 and 30 years old.

The results obtained through the application of the HUPEDCARE-Q reflect a generally moderate to low perceived level of preparedness among healthcare professionals in terms of knowledge and training related to pediatric pain management. However, this group of professionals also demonstrated high emotional sensitivity and a positive disposition toward improving practices in this area. No extreme values or significant skewness were detected in the distributions, indicating consistency in responses and an adequate understanding of the instrument by the respondents.

In this regard, there are studies that assert that observations made by healthcare professionals who interact closely with pediatric patients offer a greater perspective on the humanization of care in the management of pain through non-pharmacological treatment [43,44]. These professionals, due to the continuity of care they provide to children, are able to identify physical and emotional needs that might go unnoticed during less detailed evaluations [45,46]. Therefore, the data obtained from this questionnaire, designed to address pediatric pain and its management, are considered relevant for promoting broader use and application of this instrument, as it serves as an assessment tool that helps identify areas for improvement in the care and comfort of pediatric patients [47].

In fact, this type of instrument becomes crucial, as it facilitates interventions that significantly improve the sensory experience during painful procedures. However, to adopt meaningful changes in clinical practice aimed at the humanization of care, it is necessary for the multidisciplinary team to promote the use of validated and standardized tools that support the identification of strengths and weaknesses, thus enabling the future implementation of new guidelines and protocols [48,49].

Several studies have identified barriers to the proper management of pediatric pain, such as a lack of specific knowledge and limited training in non-pharmacological therapies. This highlights the need for continuous and specialized training in the humanization of care when addressing pediatric pain [50,51].

In this context, certain skills within clinical practice are acquired through accumulated experience, transforming clinical knowledge into a combination of theory and practice, an aspect that allows for more effective management of complex situations. Likewise, the application of new advances, such as the assessment of cortisol levels in pediatric patients, can provide objective data on pain control, improving its prevention and treatment [52]. However, it is important to recognize that healthcare professionals with longer careers may face challenges in unlearning deeply rooted myths and beliefs, which can hinder the adoption of new evidence-based practices [53,54].

Ongoing training for healthcare professionals is essential to improving the approach to pediatric pain through humanized care. Therefore, it is crucial that training programs, both in higher education and in clinical practice, address the beliefs, attitudes, and pain management practices of professionals [55,56]. In fact, this comprehensive approach helps identify entrenched practices that may interfere with the implementation of effective pain management strategies. By encouraging critical reflection on one’s own perceptions and attitudes, it becomes possible to promote more empathetic and patient-centered care, thereby improving the quality of care provided [57,58].

The validation of the HUPEDCARE-Q questionnaire content presents several limitations that should be considered when interpreting the findings. Firstly, the questionnaire is not designed to provide clinical diagnoses, rather, it serves as an exploratory tool to gather information on attitudes, beliefs, knowledge, and practices related to pediatric pain management. Since it is a self-administered questionnaire, responses may be influenced by the professionals’ subjective perception. Secondly, although the sample was large (*n* = 1.120), it was heavily concentrated in the 20–30 age group (78% of participants) and exhibited a clear gender bias (83.5% women). However, it should be noted that healthcare professions are predominantly practiced by women. The study also acknowledges that professionals with longer careers may experience greater difficulty in unlearning acquired myths and beliefs, a situation that could affect the implementation of new evidence-based educational practices. However, this hypothesis was not specifically explored, representing an additional limitation in understanding generational or educational barriers.

## 5. Conclusions

This study focused on the validation of an instrument designed to assess the attitudes, beliefs, care practices, and training of healthcare professionals regarding pediatric pain and its management through alternative treatments.

The rigorous validation of this tool is essential to ensure its reliability and validity, enabling precise identification of areas for improvement in both education and clinical practice.

Consequently, the questionnaire facilitates the development of targeted educational interventions that promote more humanized, empathetic, and patient-centered care. Systematic implementation of this instrument in healthcare settings is expected to enhance the quality of pediatric pain management, fostering improved clinical outcomes and a comprehensive approach to child care.

## Figures and Tables

**Table 1:** Content Validity Coefficient of the first and second dimensions.

Dimension	Average Response (Mx)	Individual Content Validity Coefficient (CVCi)	Factor Content Validity Coefficient (CVCf)
**Clarity**	3.76	0.94	0.92
**Pertinence**	3.78	0.94	0.93
**Relevance**	3.87	0.96	0.94

**Table 2:** Content Validity Coefficient for dichotomous questions YES/NO.

Dimension	Average Response (Mx)	Individual Content Validity Coefficient (CVCi)	Factor Content Validity Coefficient (CVCf)
**Clarity**	3.96	0.99	0.98
**Pertinence**	3.88	0.97	0.96
**Relevance**	3.94	0.99	0.98

**Table 3:** Internal consistency of three dimensions.

Dimension	No. of Items	Cronbach’s Alpha
Attitudes and beliefs	11	0.86
Knowledge and training	7	0.80
Best practices: Pediatric Pain Management	10	0.71

**Table 4:** Exploratory factor analysis of the first and second dimensions.

Items	Attitudes and Beliefs	Items	Knowledge and Training
Item 1	0.56	Item 12	0.35
Item 2	0.66	Item 13	0.83
Item 3	0.53	Item 14	0.84
Item 4	0.71	Item 15	0.51
Item 5	0.48	Item 16	0.79
Item 6	0.70	Item 17	0.80
Item 7	0.42	Item 18	0.61
Item 8	0.56		
Item 9	0.78		
Item 10	0.61		
Item 11	0.54		

**Table 5:** Exploratory factor analysis for dichotomous questions YES/NO.

Items	Best Practices: Pediatric Pain Management
Item 1	0.33
Item 2	0.51
Item 3	0.76
Item 4	0.67
Item 5	0.78
Item 6	0.51
Item 7	0.81
Item 8	0.74
Item 9	0.68
Item 10	0.49

**Table 6:** Mean and standard deviation of each questionnaire item.

Items	Sentence	Mean	Standard Deviation
Item 1	Because the neurological system is still developing in children under 2 years old, they have diminished sensitivity to pain and memory of painful experiences.	2.24	1.57
Item 2	Similar stimuli in different children produce the same intensity of pain.	1.69	1.53
Item 3	Children under 6 months old cannot tolerate opioids to relieve pain.	2.86	1.44
Item 4	After the recommended initial dose of analgesics, subsequent doses should be individualized according to the patient’s response.	4.17	1.19
Item 5	The use of non-pharmacological pain interventions is advised independently, rather than using analgesics simultaneously.	2.95	1.55
Item 6	Pain in children is a personal experience influenced by biological, psychological, and social factors.	4.02	1.24
Item 7	Non-pharmacological interventions (breastfeeding, the kangaroo mother care method, oral sucrose or glucose, and non-nutritive sucking) are very effective for mild to moderate pain control, but are rarely useful for more severe pain.	3.37	1.38
Item 8	During painful procedures, parents should not be present.	1.94	1.54
Item 9	Children in pain should be encouraged to endure it as long as possible before resorting to a pain relief measure.	1.56	1.43
Item 10	Giving children placebos (sterile water or saline, among others) is often useful to determine if the pain is real.	2.69	1.54
Item 11	Using opioids for the treatment of acute pain can cause addiction in pediatric patients.	3.47	1.29
Item 12	To verify that a child is in severe pain, changes in vital signs should be observed.	3.23	1.52
Item 13	I know and apply the scales for pain assessment in children.	3.19	1.64
Item 14	I know and apply the WHO scale of pain management levels in children (Analgesic Ladder).	3.01	1.58
Item 15	Training in acute pain in children and its management is sufficient.	2.20	1.37
Item 16	I can identify early signs of pain in newborns.	2.60	1.54
Item 17	I know how to deal with acute pain in children.	2.64	1.48
Item 18	Analgesia should be used before performing complementary traumatic tests.	3.11	1.49

Disclaimer: This is only a linguistic translation, not a validation in English.

**Table 7:** Absolute frequencies of responses to dichotomous questions YES/NO.

Items	YES Answers	NO Answers
Item 1	675	90
Item 2	708	57
Item 3	259	506
Item 4	672	93
Item 5	691	74
Item 6	722	43
Item 7	716	49
Item 8	694	71
Item 9	703	62
Item 10	226	538

## Data Availability

Data is unavailable due to privacy and ethical restrictions.

## Abbreviations

The following abbreviations are used in this manuscript:

HUPEDCARE-Q	Humanisation of Pediatric Care in Pain Management with a Non-Pharmacological Approach Questionnaire
PNKAS	Pediatric Nurses Knowledge and Attitudes Survey Regarding Pain
KASRP	Nurses Knowledge and Attitudes Survey Regarding Pain
CVC	Content Validity Coefficient
EFA	Exploratory Factor Analysis
KMO	Kaiser–Meyer–Olkin
